# A Rare Case of Escherichia coli Chest Wall Abscess With Rib Osteomyelitis in a Patient With Crohn’s Disease

**DOI:** 10.7759/cureus.13860

**Published:** 2021-03-12

**Authors:** Apaar Dadlani, Sudeepthi Bandikatla, Jennifer A Koch

**Affiliations:** 1 Internal Medicine, University of Louisville School of Medicine, Louisville, USA

**Keywords:** e. coli, chest wall abscess, inflammatory bowel disease, anti-tumor-necrosis-factor-alpha, etanercept

## Abstract

Primary chest wall abscess due to hematogenous spread is very rare and has seldom been documented in the literature, with most reported cases attributed to *Mycobacterium tuberculosis*. Prompt diagnosis and management with antibiotics, and evacuation of the abscess, is imperative as the infection can lead to systemic or disseminated infection, including erosion into surrounding bone if left untreated. We describe the case of a 67-year-old female with severe Crohn’s disease receiving anti-tumor necrosis factor-alpha (TNF-α) therapy, Etanercept presenting with localized *Escherichia coli* (*E. coli*) chest wall abscess with erosion into the surrounding rib. This case highlights a rare clinical entity, chest wall abscess, which is also an unusual site of *E. coli* infection. Only three previous cases of *E. coli* primary chest wall abscess can be found in the published literature. This case also highlights a possible association of severe Crohn’s disease predisposing to complicated soft tissue infection.

## Introduction

Primary chest wall abscess due to hematogenous spread is very rare and has seldom been documented in the literature, with most reported cases caused by *Mycobacterium tuberculosis*. Only three cases of *Escherichia coli* (*E. coli*) chest wall abscess can be found in the published literature. We describe the case of a 67-year-old female with inflammatory bowel disease (IBD) receiving anti tumor necrosis factor alpha (TNF-α) therapy, Etanercept, with localized complicated *E. coli* chest wall abscess.

## Case presentation

**Figure 1:**
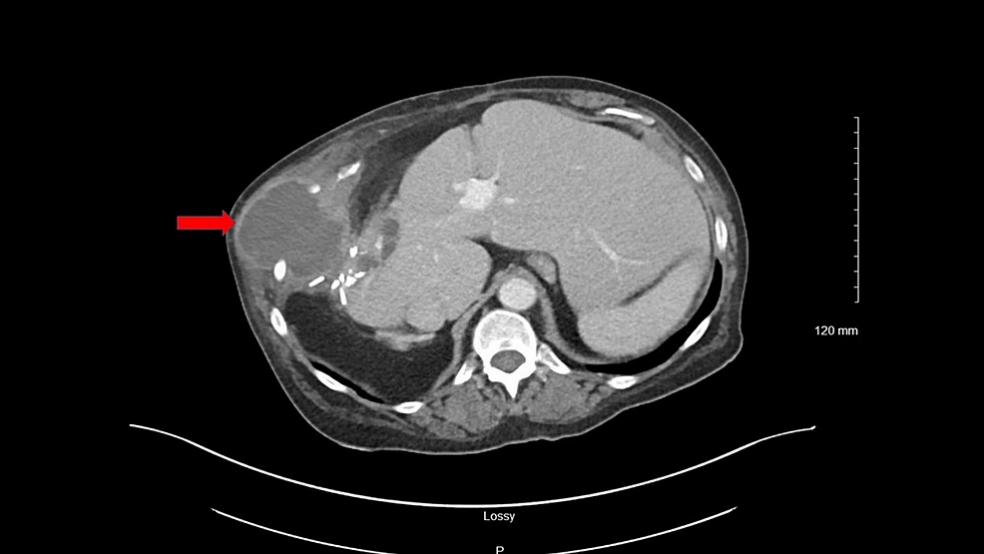
CT chest showing right chest wall abscess CT - Computed tomography

A 67-year-old female with a history of severe Crohn's disease complicated by a history of rectovaginal fistula, type 2 diabetes mellitus, and chronic kidney disease presented with fever, nausea, non-bilious non-bloody vomiting, and right lower chest and upper abdominal pain for two weeks, without a history of prior trauma. Past surgical history was significant for hemicolectomy and ileostomy for Crohn’s disease several years prior. Current medications included Etanercept injections every two weeks, atorvastatin, and insulin. Other chronic medical conditions were well controlled at presentation. On examination, she was tachycardic with a heart rate of 110/minute, a temperature of 100.6°F, and blood pressure was 110/70 mm Hg. There was a soft, fluctuating, tender mass on the right side of her lower chest and right upper quadrant abdomen. There was no punctum or drainage seen from the mass. Laboratory workup was significant for a normal white cell count of 7.0 x10^3^/µL and elevated C-reactive protein of 159.6 mg/L (reference range: ≤10 mg/L). Computed tomography (CT) chest showed a 7.5 cm encapsulated fluid collection in the right lower lateral chest wall at the level of the seventh intercostal space with erosion into the eighth rib (Figure 1). Urinalysis, urine culture, blood culture, and chest X-ray were negative for evidence of any infection. CT of the abdomen and pelvis showed the previously known rectovaginal fistula, but no evidence of perirectal or intra-abdominal abscess. Transesophageal echocardiography did not demonstrate any evidence of infective endocarditis. She underwent CT-guided abscess drainage with an aspiration of frank pus, followed by drain placement in the right anterolateral chest wall abscess. The culture of the aspirated fluid eventually grew *E. coli*. A diagnosis of *E. coli* causing chest wall abscess complicated with underlying rib osteomyelitis was made. She was started on empiric broad-spectrum antibiotics with intravenous piperacillin-tazobactam and vancomycin on admission, and she responded well to this regimen. She was eventually transitioned to oral levofloxacin for a total of six weeks of antibiotic therapy from the date of abscess drainage.

## Discussion

Chest wall abscess is a rare entity that can occur as a primary phenomenon due to hematogenous spread of infections from a different site or secondary to blunt chest wall trauma, thoracic wall surgery, direct extension from lung, or pleural infection [1,2]. Nontuberculous pathogens causing abscesses of chest wall include *Actinomyces, Salmonella, Staphylococcus aureus, Acinetobacter, Streptococcus, Prevotella bivia, Peptostreptococcus, Fusobacterium necrophorum, Burkholderia cenocepacia, Bartonella henselae, Nocardia, Entamoeba histolytica, Klebsiella ozaenae, Mycobacterium avium intracellulare*, and very rarely, *E. coli* [1,3]. To our knowledge, primary chest wall abscess due to *E. coli* has been reported in only three published cases thus far, in association with concurrent *E. coli* urinary tract infection [1,4,5]; however, only one case had evidence of bacteremia [1].

Differential diagnosis of any chest wall mass includes benign or malignant tumor, infection, or hematoma. Pyoderma gangrenosum has also been reported as a chest wall mass in a patient with IBD [6]. Diagnosis is made by CT imaging, which accurately characterizes and localizes the abscess. CT chest usually reveals bony involvement and helps in narrowing the differential diagnosis [7]. The severity can range from a few symptoms with systemic signs of infection to life-threatening situations that require urgent and aggressive care. The mainstay of treatment of primary nontuberculous chest wall abscess includes antibiotic therapy and prompt drainage. In some cases, complex reconstructive surgeries may be required [2,8,9].

IBD increases the risk of invasive bacterial infection by alteration of the barrier function in the inflamed intestinal wall leading to increased permeability and malnutrition-associated immune dysfunction. Additionally, biological agents used to treat IBD such as anti-TNF-α therapeutics have been found to be independently associated with a variety of infections due to a decrease in the immune system's response [10,11]. In a study on 5,522 patients with IBD, Goren et al. reported that 1.3% of patients had bacteremia; these patients were mostly above the age of 65 years, and the most common pathogen was *E. coli* [10]. In our patient, a thorough investigation did not reveal an obvious source of bacterial seeding. Though few cases of costochondritis due to *E. coli* infection have been published [1,5], the lateral location of the chest wall abscess in our patient made it less likely to have originated as costochondritis, which is usually parasternal. It has been established that bacterial translocation in Crohn’s disease is not uncommon, and 29% of patients in a study by Laffineur et al. had evidence of bacterial translocation, of which the most common pathogen was *E. coli* [12]. We believe that our patient’s severe Crohn’s disease with fistula, along with immunosuppression with Etanercept, may have resulted in *E. coli* translocation from the intestinal wall, followed by transient bacteremia and seeding of the chest wall, ultimately resulting in chest wall abscess.

## Conclusions

Our case highlights a rare clinical entity, chest wall abscess, which is an unusual site of *E. coli* infection. Prompt diagnosis and management with antibiotics and evacuation of the abscess are imperative as the infection can lead to systemic or disseminated infection including erosion into surrounding bone if left untreated. This case also highlights a possible association between severe Crohn’s disease and the predisposition for complicated soft tissue infection. Further observation and study of this association is needed as the options for immunosuppression in patients with IBD continue to expand.
